# The problem of utilization (detoxification) unsymmetrical dimethylhydrazine. Challenge for synthesis of mildronate and its structure analogues

**DOI:** 10.1186/1878-5085-5-S1-A92

**Published:** 2014-02-11

**Authors:** Vadym G Voloshchuk, Yuriy L Yagupolskyi, Yuriy V Tanchuk, Volodymyr M Boyko, Rostyslav V Bubnov

**Affiliations:** 1 Institute of organic chemistry, National Academy Sciences of Ukraine, Kyiv, Ukraine; 2 Clinical Hospital “Pheophania” of State Affairs Department, Kyiv, Ukraine

## Background

Sustainable development requires that hidden environmental costs of technologies, that should be paid by future society, to be taken into account. Scientific methods and institutions have tended to emphasize the study of individual natural processes rather than systems, analysis more than synthesis, and understanding nature more than predicting its behavior. Science of Medicine of future is increasingly being called on to produce knowledge and technology that promote environmentally sustainable, people-oriented development and long-term management of resources. *Unsymmetrical dimethylhydrazine* (UMDH) has extremely toxic (1^st^ class of danger) and highly carcinogenic properties [1,2]. UMDH was and still is a high quality liquid rocket fuel, and remains to be indispensable for the latter stages of ballistic missiles and space. After 1992 about 5000 t of UMDH in disarmament Ukraine and more than 80,000 t in Russia have accumulated over the years of the Cold war, creating an extremely serious threat to the environment and all living being. Therefore, the problem of complete and irreversible UMDH destruction (utilization) can be solved either by complete destruction or to use UMDH as source for the production of chemical products, medication (e.g., mildronate and its structure analogues). The problem of utilization (detoxification) UMDH was extensively discussed in [1].

## The aim

is to develop and implement the own cheap and effective method of detoxification of unsymmetrical dimethyl hydrazine for the medical needs.

## Technological solution

The first phase includes utilization of unsymmetrical dimethylhydrazine for chemical synthesis of mildronate and its structure analogues in specialized chemical laboratory. In turn, we made a number of demands to the chemical structures, which can be used as UMDH acceptors and discovered a new type of chemical interactions of heptyl and its derivatives, suggesting that: after relevant studies the chemical recycling methods of heptyl can be greatly expanded, including the medical aspect. In all cases, the implementation of this idea is based on the known chemical properties of UMDH *and finding new ways of its transformation*. Materials about the new chemical interactions of heptyl we have not still published.

## Conclusion

Through the use of UMDH in organic synthesis to create harmless and promising for practical application products and thereby solve the problem of its complete destruction.

## Outlook and expert recommendations

It is recommended to create a work group on the basis of institutions and research centers of the National Academy of Sciences of Ukraine, authorized to carry out such works with heptyl. To involve to the project EU research institutions, pharmaceutical industry for scientific and financial support. The estimated initial staffing of specialized laboratory is about 4-5 experts. We suggest tocarry out the initial stage of study of the pharmacological properties of the obtained materials at the Institute of Pharmacology and Toxicology Ministry of Health of Ukraine. The mean duration of the dismantlement of heptyl to the form of medical structures is 5-10 years. Further work in this field depends on the obtained results, as well as a number of factors that are unpredictable at present.
